# Policing the profession? Regulatory reform, restratification and the emergence of Responsible Officers as a new locus of power in UK medicine

**DOI:** 10.1016/j.socscimed.2018.07.042

**Published:** 2018-09

**Authors:** Marie Bryce, Kayleigh Luscombe, Alan Boyd, Abigail Tazzyman, John Tredinnick-Rowe, Kieran Walshe, Julian Archer

**Affiliations:** a Collaboration for the Advancement of Medical Education Research and Assessment (CAMERA), Faculty of Medicine and Dentistry, University of Plymouth, Plymouth, UK; b Alliance Manchester Business School, University of Manchester, Manchester, UK

**Keywords:** UK, Medical profession, Regulation, Revalidation, Restratification, Responsible Officers, Qualitative, Survey

## Abstract

Doctors' work and the changing, contested meanings of medical professionalism have long been a focus for sociological research. Much recent attention has focused on those doctors working at the interface between healthcare management and medical practice, with such ‘hybrid’ doctor-managers providing valuable analytical material for exploring changes in how medical professionalism is understood. In the United Kingdom, significant structural changes to medical regulation, most notably the introduction of revalidation in 2012, have created a new hybrid group, Responsible Officers (ROs), responsible for making periodic recommendations about the on-going fitness to practise medicine of all other doctors in their organisation.

Using qualitative data collected in a 2015 survey with 374 respondents, 63% of ROs in the UK, this paper analyses the RO role. Our findings show ROs to be a distinct emergent group of hybrid professionals and as such demonstrate restructuring within UK medicine. Occupying a position where multiple agendas converge, ROs' work expands professional regulation into the organisational sphere in new ways, as well as creating new lines of continuous accountability between the wider profession and the General Medical Council as medical regulator. Our exploration of ROs' approaches to their work offers new insights into the on-going development of medical professionalism, pointing to the emergence of a distinctly regulatory hybrid professionalism shaped by co-existing professional, managerial and regulatory logics, in an era of strengthened governance and complex policy change.

## Highlights

• Responsible Officers are a new governance elite group in the UK medical profession.
• They work at the nexus of professional, managerial and regulatory spheres.
• Differ from other doctor-managers due to accountability for medical performance.
• Organisational context shapes experiences of this new role.
• Regulatory reform has engendered a new form of hybrid professionalism.

## 1 Introduction

Doctors' work and the changing, contested meaning of medical professionalism have long been a focus for researchers, and in recent years the medical profession's place in relation to reconfigured models of healthcare management and governance has generated extensive interest. Much attention has centred on those doctors working at the interface between healthcare management and medical practice, and has demonstrated that such ‘hybrid’ doctor-managers provide valuable analytic material for exploring changes in how medical professionalism is understood (Kuhlmann et al., 2013; McGivern et al., 2015; Noordegraaf et al., 2016; Waring, 2014). Such work has recognised, amongst some international commonalities, the importance of significant national specificities, particularly when analysing relationships between professionals and states or organisations (Bezes et al., 2012). Here, we seek to add to such critiques by exploring the implementation of regulatory reforms in the United Kingdom (UK), which have, for the first time, placed considerable statutory powers and duties in the hands of a nominated medical professional in each organisation employing or contracting with doctors, formally titled the ‘Responsible Officer’ (RO).

First, we set out the background and context to this development, describing in overview the nature of the reforms leading to these changes. We then draw on theories of professional restratification, Foucault's concept of governmentality (Foucault, 1991), and research on hybrid professionals to frame our analysis of the RO role, with a particular focus on their responsibility for the implementation of medical revalidation, a new regulatory mechanism in place since 2012. Using this theoretical framework, this paper analyses qualitative data from a national survey of ROs, and discusses the insights these new hybrid professionals offer for understanding professional responses to regulatory reform.

### 1.1 Regulatory reform and the medical profession

The creation of the RO role, and the introduction of revalidation, notably changed how medical practice in the UK is regulated; the latest in a series of policy shifts affecting the governance of the medical profession. Historically, medicine operated a model of self-regulation both formally and informally, at group and individual levels (Chamberlain, 2009). Practitioners were expected to regulate themselves by practising in accordance with shared professional standards (Waring, 2007). Since 1858, the General Medical Council (GMC) has controlled professional registration, and assured standards of medical education. Through its Fitness to Practise (FTP) procedures, the GMC investigates allegations of poor performance or misconduct. However, traditionally much management of poor performance occurred locally and informally, relying on collegiate discussions and ‘in-house’ resolution rather than formal regulatory mechanisms (Rosenthal, 1995). Moran (2003) characterised this as ‘club regulation’, a lasting expression of the Victorian regulatory state, focused on maintaining good relations within the profession. The profession was thus entrusted with regulating its membership by the state and society, in a ‘a neat and powerful arrangement’ (Salter, 2001).

Latterly, however, this arrangement has altered dramatically, with a move towards bureaucratic regulatory oversight (Waring et al., 2010), pointing to some erosion of professional autonomy (Dixon-Woods et al., 2011), and the profession no longer solely responsible for its own regulation. Broad consensus exists on the contributing factors that converged to politicise medical regulation and create an appetite for change. First, since the 1980s, successive governments' adoption of neo-liberal New Public Management (NPM) principles extended state interest in healthcare delivery and organisation, and consequently in monitoring clinical standards (Waring et al., 2010). Concurrently, emergent patient groups (Mold, 2010) contributed to scepticism about medical authority (Salter, 2001). Finally, high profile malpractice scandals in the 1990s and 2000s raised doubts about the profession's ability to self-regulate effectively (Dixon-Woods et al., 2011; Salter, 2007; Waring, 2007). Consequently, in the 2000s, the GMC was reconstituted to reduce medical dominance, and gained powers to oversee not just professional misconduct but poor performance.

The changed political mood added impetus to long-mooted plans for revalidation (Archer et al., 2015), accompanied by the creation of the RO role, whose origins lay in GMC proposals that revalidation should entail local certification of doctors' participation, by an organisational representative, such as the Medical Director or Chief Executive (Smith, 2004). Following the Shipman Inquiry's criticism of GMC plans, strengthened new proposals, more clearly defining the responsibilities associated with local assurance of revalidation, and assigning these to a specific new RO role, were set out by the Chief Medical Officer (Department of Health, 2008). Subsequent legislation (Health and Social Care Act, 2008) required organisations employing or contracting with doctors to appoint an RO before revalidation was introduced in 2012.

### 1.2 Responsible Officers and regulation

Revalidation aims to monitor doctors' fitness to practise throughout their careers. Comparable schemes exist or are under consideration internationally (Boulet and van Zanten, 2014; Sehlbach et al., 2018), marking a notable trend towards continuing assessment of competency. However, the RO role is a striking feature of the UK medical regulatory system, when compared to others internationally (Archer and Regan De Bere, 2013).

Revalidation requires doctors to document their practice and participate in annual appraisals (General Medical Council, 2012). Their RO then brings appraisal information together with other clinical governance data to make a formal recommendation to the GMC, usually every fifth year (General Medical Council, 2015). ROs may recommend that doctors be revalidated, or that their revalidation be deferred, or notify the GMC that the doctor has not engaged. Using this recommendation, the GMC decides whether to renew the doctor's licence to practise.

Greenhalgh and Wong (2011) described the revalidation process as essentially technical and bureaucratic, aligned with scientific-bureaucratic medicine, including increased managerialism. Its introduction was contested from within the profession (Archer et al., 2015), due to fears of its reductive impact on professional autonomy and the challenge of reconciling formative appraisal processes with a summative regulatory mechanism (Archer et al., 2017). Subsequently, amongst those in leadership positions at least, previously conflicting discourses of professionalism and regulation have converged, driven by the legislative imperative to implement the policy (Tazzyman et al., 2018). However, positioning revalidation as a policy move from embodied trust in professionals to state enforceable trust, Spendlove (2018) identified continued professional resistance manifested in doctors' formalistic approaches to engagement.

The approximately 600 ROs are intrinsic to this regulatory process and must also monitor the fitness to practise of doctors connected to their organisation (The Medical Profession (Responsible Officers) Regulations, 2010). They work for organisations ranging from those with just a few connected doctors to those with several thousand, across NHS, independent and third sector settings (NHS England, 2016). In most cases, the role is held by the Medical Director (MD) or Deputy Medical Director. Some, often smaller, organisations contract out the role, and some ROs fill the role for multiple organisations.

### 1.3 Interpreting professional responses to regulatory reform

Existing research on ROs has typically focused on the practicalities of their work, particularly during early implementation (Nath et al., 2014; Shepherd and Cameron, 2010; Webster and McLachlan, 2011), or on their own experiences of undergoing appraisal (Furmedge et al., 2016; Griffin et al., 2015). In this paper, we analyse the RO role in the light of theoretical interpretations of comparable hybrid doctor-manager groups, to better understand their position at the interface of this fundamentally changed relationship between medical regulation and healthcare organisations.

In some quarters, the curtailment of professional self-regulation has been seen, alongside increased managerial scrutiny of medical work, as having fundamentally undermined professional autonomy, as part of an international trend of ‘deprofessionalization’ (Bezes et al., 2012; Schlesinger, 2002). The diffusion of NPM principles brought an expansion of non-medical management in healthcare and new systems of performance management and financial control of medical practice (Ackroyd et al., 2007). However, as Le Bianic (2012) notes, analyses focusing solely on reduced autonomy position professionals as ‘passive agents of reform’ and ‘frontally opposed’ to managerialism. Alternative analyses have foregrounded more active professional responses to this changed political and social environment. Particular attention has focused on the emergence of clinical managers as new professional elites operating at the intersection between the medical profession and organisations (Cascón-Pereira et al., 2016; Correia and Denis, 2016; Kuhlmann et al., 2013; Martin and Waring, 2013). Such ‘hybrids’ (McGivern et al., 2015) offer insights how the medical profession has responded to regulatory and organisational reforms. Theoretical interpretations have centred on two concepts: restratification and governmentality.

Developed in response to the perceived threat of deprofessionalization, the restratification thesis (Freidson, 1985, 1994, 2001) posited that elite groups would operate oversight and control over the mass, or ‘rank and file’, of their profession. For Freidson, professionalism was a ‘third logic’ by which professional work may be controlled, existing alongside and in competition with economic market forces and managerial bureaucracy (Freidson, 2001), and it was through the internal restructuring of restratification that collective autonomy would be maintained. Professional elites would mediate between the profession and the state, working to safeguard professional interests (Chamberlain, 2013; Waring, 2014).

Freidson's delineation of restratification identified a ‘knowledge elite’, focused on professional education, research and setting professional standards, and an ‘administrative elite’, responsible for managing professionals' work activity. Importantly, the retention of professional autonomy is linked to recognition that expert knowledge is requisite for some activities, restricting some roles to those from within the profession (Freidson, 2001). The RO role is one such function, reserved for those holding medical qualifications, though this may be as much to maintain regulatory oversight over role-holders as any need for medical expertise.

Restratification has been used to understand changes in the medical profession (Chamberlain, 2014), and particularly the position of doctor-managers, though applied analysis has highlighted the need for nuanced understanding of how professional strata manifest with specific settings (McDonald et al., 2009). Waring (2014) has further developed Freidson's categories, suggesting, amongst other variations, that the ‘administrative elite’ may now be better viewed as encompassing subgroups – a ‘managerial elite’ and a ‘governance elite’. This revised categorisation offers a more refined framework in which to place new professional roles, including ROs. Specifically, Waring (2014) identifies the ‘governance elite’ as ‘a growing professional stratum that reflects the sub-specialisation of managerial elites’, with responsibility for monitoring and safeguarding professional standards, and functioning as a link to external regulatory bodies and organisational management.

Often discussed alongside restratification, governmentality (Foucault, 1977, 1991) holds that adopting externally imposed standards may engender their internalisation, whether by individuals (Waring, 2007) or the profession, acting as a conduit for state ‘governance’ power (Chamberlain, 2013). This internalisation of external expectations constitutes self-surveillance, reducing or negating the need for further external oversight (Exworthy, 2015). Applying this interpretive lens positions managerial or governance elites as those operating surveillance over their peers (Waring, 2014), and has clear relevance for ROs, who fulfil legislative requirements to oversee medical performance as part of a regulatory system. The surveillance of doctors' performance is thus still situated within the profession, though the regulatory drivers for the surveillance are ‘owned’ by the GMC and aligned to state aims (Waring, 2007).

Interpretation of professional elites' activities as evidencing restratification, governmentality, or some combination of these two abstract theses typically rests on investigating how role-holders self-identify and behave. McGivern et al. (2015) found distinct patterns amongst doctor-manager hybrids, distinguishing ‘incidental hybrids’ from ‘willing hybrids’, where ‘willing hybrids’ had assimilated organisational priorities and actively sought to develop the managerial aspects of their work and identities. Such willing hybrids may be seen as embodying ‘organisational professionalism’ (Evetts, 2009), working within organisational hierarchies and systems for the control of professional work. Research into the identity work of hybrid professionals has highlighted that identity formation in doctor-managers is strongly influenced by contextual and institutional factors (Cascón-Pereira et al., 2016; Currie et al., 2009; Correia and Denis, 2016). Organisational context has been identified elsewhere as an important element in shaping how ROs' make revalidation recommendations (Webster and McLachlan, 2011).

Hybrid professionals have also been presented as representing negotiation and convergence between professional and managerial cultures (Numerato et al., 2012), and as working across and between both domains rather than as maintaining or adopting a singular position on one side of a binary divide (Noordegraaf et al., 2016). In such cases, managerial and professional discourses overlap, as hybrid professionals serve multiple agendas simultaneously and sometimes ambiguously (Olakivi and Niska, 2017). In this way, professional work is multi-dimensional and may be shaped, within a specific context or setting, by ‘co-existing logics’ which may be competing or convergent (McDonald et al., 2013).

Professional, managerial and regulatory logics, and linked discourses, have been identified in relation to revalidation (Archer et al., 2015; Greenhalgh and Wong, 2011; Tazzyman et al., 2018). We therefore draw upon the abstract theoretical frameworks offered by existing restratification and governmentality-informed perspectives on professional hybridity to underpin an applied analysis addressing two connected questions. Firstly, we ask whether ROs constitute a distinct professional elite group and, secondly, we explore whether role-holders undertake their work in defence of profession autonomy, in pursuit of organisational or regulatory priorities, or operate through an integrative hybrid professionalism.

## 2 Methods

This paper presents an analysis of qualitative data collected through a UK-wide survey of ROs, from which overview findings are reported elsewhere (Walshe et al., 2017). The survey, conducted online between July and September 2015 using Qualtrics (2015), was distributed to 595 ROs as part of a wider study evaluating medical revalidation and its impacts on organisational performance and medical practice. Ethical approval for this study was awarded by the University of Manchester ethics committee (REC 15028).

Numeric, rating scale and free text data were collected, focusing on four key areas:

• Individual, organisational and external resources for revalidation;
• Organisational systems for managing medical performance;
• How revalidation recommendations to the GMC are made;
• The implementation and impact of revalidation.

Responses were received from 374 ROs (63%), though responses were lower from ROs for organisations with less than 20 doctors and locum agencies at just under 50%. A majority of respondents (64%) held RO and Medical Director roles concurrently, six percent were RO and Deputy Medical Director, and seventeen percent held other senior management roles. Initial analysis of the survey data included thematic analysis of the free text data, using a template analysis approach (King, 2012). Using Dedoose (2016) web application for managing and analysing qualitative and mixed methods research data, a coding framework was developed inductively from 40 respondents' comments by five researchers (MB, KL, AB, AT, JTR). The initial framework was discussed and revised, and the resultant coding template was applied to all the free text data by three researchers (KL, AT, JTR). Codes were drawn together into themes through discussion amongst the research team. In this initial analysis, ‘experiences of the RO role’ was identified as a key theme, with respondents discussing the nature of their role, both in response to questions asking specifically about their revalidation decision-making practices but also in response to other survey questions.

Recognising ROs as holding a key role in medical regulation, we undertook the in-depth theoretically-driven analysis of this themed data reported here. In this analysis, MB and KL revisited the data relating to ROs' experiences of and reflections on their role, and identified nuanced subthemes about ROs' work and perspectives. This involved moving iteratively between the data and the theoretical literature outlined above to refine our analysis, drawing on the varied disciplinary expertise of the wider team, including medical education, sociology, and organisational research. Such an approach has previously been used successfully to develop theoretical contributions from existing empirical datasets (Martin et al., 2015). Reporting qualitative survey data separately has also been demonstrated to be an effective way to explore views across a population of interest (Dale et al., 2016) and as providing adequate data to support theory driven interpretations of professional activities (Entwistle and Matthews, 2015). In this case, the high response rate overall resulted in broad coverage across a cohort little studied elsewhere, and provided sufficient content to support this exploratory analysis of the RO role. However, the lower response rate from ROs in smaller organisations and locum agencies may mean these perspectives are less well represented in our analysis. As not all respondents provided responses to all free text questions, raising questions of non-response, we have not sought to generalise our findings, but have focused on emerging concepts.

We present our findings with illustrative quotations and information about respondents' job roles and organisational type to provide context, broadly categorised to maintain anonymity as ‘NHS secondary care’, for example, or ‘independent provider’ for private, for-profit healthcare organisations.

### 2.1 Findings

Our findings centre on three themes: firstly, the RO function, professional hierarchy and hybridity; secondly, ROs' regulatory decision-making and professional accountability; and finally, ROs' reflections on the role and its impacts.

### 2.2 ROs, professional hierarchy and hybridity

Firstly, we sought to understand whether ROs form a distinct elite group within the professional hierarchy of UK medicine. Within the data, 109 ROs made comments about their role relating to their disciplinary power. Such comments often emphasised the separation between them and the doctors under their authority:

*…The doctors do regard the RO as someone to keep in with. I am seen as firm but fair and helpful. Dealing with some difficult cases with compassion and realism leads to wider respect in the community. Being very strict with some game-players leads to a serious ethos in the Trust.* (RO53, RO, NHS Secondary Care)

As in this instance, many ROs discussed themselves and their work from the perspective of others, describing how they thought others perceived them. Here the RO function is presented as having enhanced and strengthened the MD role, which a majority of respondents also held:

*…some slightly greater engagement with doctors but I think they all now associate the Medical Director with being a policeman/headmaster/oppressor.* (RO381, RO/Medical Director, NHS Secondary Care)

*…Doctors feel they are more dependent on me, as they think their professional fate is in my hands (slightly wrong perception).* (RO222, RO/Medical Director, NHS Mental Healthcare)

Adopting the perspective of the ‘other’ in this way emphasises the relational nature of stratification in the profession (Waring, 2014), where groupings are identified as much by their relationships to and differences from each other, as by internal characteristics. We found evidence, therefore, that ROs see themselves as set apart from other doctors. Notably, respondents used terminology imbued with notions of power and authority, invoking explicitly hierarchical ideas of oversight and discipline.

Occasionally, respondents offered reflections about the role and the potential for some ROs to wield this authority unfairly by persecuting doctors:

*Some ROs seem to be on a mission to weed out bad doctors, or those they consider to be bad doctors, and have a lot of power - the system is open to prejudice (some of which I have seen). I don't know what can be done about this. The role of RO might attract the type of individual who wishes to judge others …* (RO376, RO/Clinical Director, Independent provider)

We also found that respondents commented on differences between their prior experience of medical management and being an RO, identifying the advent of the RO role as a change. With ROs having identified that their oversight role may arouse apprehension amongst other doctors, and that the powers they hold have the potential to be misused, some respondents felt unease about the role, though the reasons for this unease differed:

*I became RO after revalidation was introduced. It gives the MD a lot of (unwelcome) power.* (RO271, RO/Medical Director, NHS Secondary Care)

[Being RO] *Has raised my profile and generally heightened my awareness of issues around professionalism. I also have a greater sense of potential for personal exposure, which is not particularly pleasant …* (RO215, RO/Deputy Medical Director NHS Secondary Care)

Again, RO work is presented as a change from the MD role with the distinctly regulatory nature of ROs' responsibilities bringing an added dimension to this new hybrid group's work.

We also found less direct allusions to oversight, with respondents commenting on the increased leverage the role offers to bring about doctors' compliance with expected standards. The application of authority was seen in references to ROs' ability to drive up standards through quality improvement and performance management:

*I think that the RO role gives us an important quality improvement tool …* (RO163, RO/Medical Director, NHS England Area Team)

*Probably more ‘strict’ about encouraging professionalism amongst all doctors. I feel that it has given me a useful ‘lever’ to encourage doctors to improve performance …* (RO33, RO/Medical Director, NHS Secondary Care)

Within the data pertaining to power and hierarchy, we found 61 respondents made allusions to a regulatory discourse of performance management, focused on individual doctors' adherence to standards (Archer et al., 2015). We also identified an equivalent number of respondents (n = 53) who referred to a quality improvement discourse, previously identified elsewhere as used by managerial elites (McGivern et al., 2015).

We found further evidence of managerialism in ROs' comments noting that they had been able to focus their colleagues on wider organisational goals:

*…RO formal responsibility has helped focus the mind of all staff and the board on quality.* (RO308, RO, Independent provider)

*I have become more central to the work of all the doctors at* [organisation name] […] *There is a sense that the doctors in the organisation, with me as their representative and RO, are more aligned to the organisational goals than previously.* (RO109, RO/Medical Director, Mental health charity)

These respondents felt that the RO role had impacted on medical work within their organisations, and whilst these ROs were based in non-NHS organisations, we found allusions to quality improvement and organisational agendas from across healthcare settings:

*I now have a much stronger grip on the performance and conduct of doctors, and have been able to use the needs of appraisal and revalidation to bring about a number of quality improvements that have benefitted the whole organisation.* (RO241, RO/Medical Director, NHS Community)

This RO was one of sixteen who referred to both a regulatory discourse of performance management, and the managerialist focus on quality improvement in their comments demonstrating how these two logics can converge in RO work.

In these instances, ROs are consciously using their position of authority to encourage doctors to focus on particular organisational priorities. This convergence of discourses (Olakivi and Niska, 2017) demonstrates ROs' significance both within organisational management structures, as well as within the professional hierarchy, whilst also emphasising the novel regulatory dimension of their hybridity.

### 2.3 Regulatory decision-making and professional accountability

With many ROs identifying themselves as having a distinct position and authority over other doctors in their organisations, we found the specifically regulatory character of their work foregrounded in comments from 117 respondents relating to their revalidation decision-making responsibilities. Our findings in this domain shed further light on how ROs operate to fulfil their regulatory responsibilities, meet organisational priorities, and navigate the multi-faceted nature of their work.

Discussing operational aspects of RO work, some expressed trepidation about their responsibility for judging other doctors' performance, or general apathy from doctors:

*It's* [revalidation] *complicated and doctors just don't get it and they have no respect for it. Hard slog to engage them, my team are often in the firing line …* (RO307, RO/Medical Director, Independent provider)

Whilst many respondents had made deferrals or dealt with concerns about individual doctors, in a small number of cases ROs reported having been brought into conflict as a result of their decision-making:

*I have had an issue with an individual where a deferral was required who threatened to resign and leave if I did not change my opinion* […] *it created a significant tension between myself and the CEO who was focused on the bottom line.* (RO125, RO/ Medical Director, Independent provider)

Whilst not common, such comments do demonstrate the potential for ROs to face defensive reactions from individual doctors, but also that there may be limitations upon ROs' authority within their organisation. The priorities of an organisation's executive leadership may at times conflict with regulatory demands, perhaps especially in an independent organisation focused on revenue.

Although the majority of respondents appeared relatively satisfied with the process of making recommendations, others sounded notes of caution, highlighting the varying degrees of knowledge ROs might have about the doctors concerned:

*It relies on knowledge of the individual doctor and their reputation as well as the appraisal evidence. I would be reluctant to revalidate a doctor I don't know in this way.* (RO308, RO, Independent provider)

*I work across a multi-centre organisation, and often have never met my doctors even* [when] *making recommendations …* (RO307, RO/Medical Director, Independent provider)

Such comments show the importance of organisational context, particularly organisational size, in shaping ROs' experiences of the role, and also suggest that the information available to them may differ considerably. These comments echo findings from early work on revalidation which highlighted the variety of ways in which revalidation decisions could be made (Webster and McLachlan, 2011).

The legislation associated with revalidation assigns accountability for monitoring medical performance to the RO personally, rather than to healthcare organisations corporately. Where comments within the data pertaining to decision-making addressed this issue directly, ROs were divided about whether they should be solely responsible for making revalidation recommendations, or whether that task should be shared or delegated. Thirty-three respondents particularly stressed the importance of personal responsibility:

*The workload involved in making the recommendations is large and I currently don't have the confidence to delegate this to anyone else (probably quite rightly).* (RO77, RO/Medical Director, NHS Secondary Care)

Conversely, 37 respondents, mostly although not exclusively working in organisations with larger numbers of doctors, such as secondary care and mental health hospitals, specified that they had established decision-making groups to assist in making recommendations. One went so far as to argue that in large organisations, the RO role should be abolished and accountability transferred to a team:

*…the job of RO is virtually impossible to do well - and ends up being too reliant on quality of appraisal, which is variable … I would take away the single RO post completely and make the job a responsibility of the medical governance team …* (RO376, RO/Clinical Director, Independent provider)

Though we cannot generalise from the proportions within the data, these data do show that ROs' shared regulatory responsibilities can be experienced in varied ways, and that variation may be due to organisational context or size, or to ROs' individual preferences.

The authority of the RO role brings with it significant responsibility, and responses to this are mixed with some tensions arising from this evident in our data. The RO embodies this regulatory responsibility in a wholly new way, and the decision-making about other doctors' performance required by revalidation differentiates their work from that of other doctor-managers.

### 2.4 Becoming ‘responsible’: reflections on regulatory work

The impact that doing the regulatory work of an RO had on respondents was a major theme within the data, with both positive and negative changes identified. Workload was discussed by 114 respondents to the survey and, whilst not surprising as the survey focused on the impacts of implementing revalidation, within this data we identified comments highlighting that ROs' regulatory work was often an addition to other roles. Only eleven respondents stated that the impact on their workload had been minor, and almost all of those worked for organisations with small numbers of connected doctors. The majority of comments noted that RO work had added burden to their working lives:

*It has taken up a lot of time. Other things in my portfolio have not been given full attention as a result.* (RO367, RO/Deputy Medical Director, NHS Secondary Care)

*It has introduced a significant amount of additional administration and takes up time that would be far better spent doing the job I am employed to do.* (RO237, RO, Medical defence organisation)

The latter respondent here appears to regard the RO role as imposed upon them, contrasting it with their other work, though they did not give information about their other work. Individuals working as ROs can often hold the role concurrently with other roles, be that the MD role as for the majority of survey respondents, or continued clinical practice, for instance. This brings additional complexity to notions of professional hybridity, as acquiring managerial or governance responsibilities does not mean that other working commitments are necessarily set aside. Our data suggest that balancing multiple roles brings practical challenges of time and workload, and may also create tension in terms of identity formation or fluidity.

Forty-eight respondents indicated that assuming the RO role had increased their professional standing within organisational hierarchies and brought greater co-operation with colleagues due to the extension of formal governance processes embedding revalidation into wider management structures:

*The RO role has assisted in much closer and better working with medical leaders and the Board/ across the organisation.* (RO117, RO/Medical Director, Independent provider)

*I have respect from the team and the CEO for the decisions I have made on the doctors so far.* (RO195, RO, Locum agency)

Again, the relational nature of RO identity work is evident. Whereas ROs described their power and authority in reference to other doctors, here they refer to their status in organisational management structures.

Less frequently, respondents reflected on their own personal development:

*As it affects me personally, more transparency about my own performance; I've done more CPD; I've sought evidence about my outcomes; ensured mandatory training done, etc …* (RO295, RO/Deputy Medical Director, NHS Secondary Care)

*I have developed increased resilience. I have learned a lot from other ROs in NHS England … I have realised that we need to harness the leadership skills of several layers of medical management to get the job done because it is so massive one person can't do everything.* (RO148, RO/Medical Director, NHS England Area Team)

In this instance, the respondent positions themselves at the apex of a hierarchy of medical management within an organisation, and cites the importance of interaction and knowledge exchange with their RO counterparts. Others too identified interaction with RO peers as having been beneficial. In particular, there were references to RO network meetings, organised by NHS England to bring ROs together to discuss best practice and share learning:

*The Regional and National Network meetings are by far the best forum for learning, developing and being supported. They are absolutely essential in my opinion. (RO355, RO/Medical Director, Independent provider)*

Of the seventy-four ROs who mentioned network meetings, all but four were positive about their value, though a few noted a preference for regional or sector-specific meetings over large national-level gatherings.

Organised network meetings bring ROs together as colleagues, in a way which seems to positively reinforce their sense of being a distinct occupational group, contributing to the formation of a specifically ‘RO’ group identity, as distinct from the broader cadre of doctor-managers. Moreover, the existence of formalised RO to RO information-sharing channels (Revalidation Support Team, 2013), designates the RO as the conduit for medical performance related exchanges between organisations. In this way, as well as sitting at the intersection between an organisation and the professional regulator, doctors holding the RO role may form a new web of interactions across organisational boundaries.

## 3 Discussion

Regulatory reform, particularly the advent of revalidation, has decisively changed the way oversight of the UK medical profession operates (Archer et al., 2017; Tazzyman et al., 2018). This has variously been seen as an extension of bureaucratic-scientific medicine (Greenhalgh and Wong, 2011) and an increase in governmental control (Chamberlain, 2014). Within this changed environment, ROs hold a key position, entrusted with evaluating the performance of their medical colleagues, and providing a link between medical work in organisational settings and the national professional regulator. The findings presented in this paper demonstrate firstly that ROs' responsibility for monitoring the performance of other doctors within organisations has altered the professional hierarchy, entrenching a divide between ROs as a ‘governance elite’ group (Waring, 2014) and the ‘rank and file’ doctors subject to their oversight.

In terms of professional structure, we found evidence that ROs both distinguish the RO role from other managerial work, and that they describe the position of the RO in relation to other groups (Waring, 2014). Notably, ROs typically described themselves as set apart from and above the doctors whose performance they oversee, and explicitly characterised the relationship between themselves and other doctors in their organisation in terms of their own authority within that dynamic. The regulatory decision-making tasks central to the RO role therefore both alter power dynamics within the profession and give a distinctive regulatory, or governance, character to this elite group.

ROs also positioned themselves in relation to local healthcare management structures, highlighting that their regulatory responsibilities are conducted in particular organisational contexts. In common, therefore, with purely managerial roles, the RO function is organisationally situated, and experiences of the role are necessarily shaped by the nature of the organisation, echoing findings from earlier studies (Nath, 2013; Webster and McLachlan, 2011). Our findings show that ROs' regulatory decision-making about doctors' performance is mediated by organisational context, with organisational size for example, impacting on the burden of work, and the amount of information available about doctors varying between settings.

**Fig. 1:**
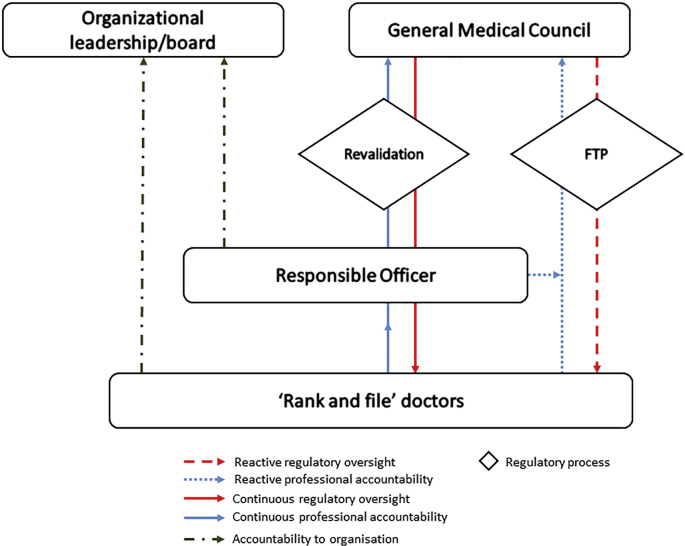
The RO as nexus between organisation, profession and regulator.

The RO has come to embody accountability for medical performance within organisations, balancing authority over rank and file doctors with their responsibilities to the GMC as external regulator. Fig. 1 sets out lines of regulatory oversight and professional accountability which position ROs above other doctors in their organisations in a professional hierarchy within their organisational context, but also show that the GMC exerts its regulatory power both over ROs themselves, and through them over the profession as a whole. Revalidation, as on-going process encompassing all doctors, has strengthened regulatory oversight, with ROs being the nexus between the organisational, regulatory and professional spheres.

Our study sheds light on how ROs undertake their work, identifying that some ROs draw upon a quality improvement agenda underpinned by a managerialist logic, or see their role as being to improve doctors' performance in order to promote professionalism. This latter discourse appears to emanate from a regulatory logic focused on remedying apparent performance deficits (Archer et al., 2015). A similar mix of discourses has been identified elsewhere in work focused on doctor-managers (McGivern et al., 2015), and suggests that where professionals take on managerial or governance responsibilities, they can draw upon different motivations, though these may overlap or converge (Noordegraaf et al., 2016; Olakivi and Niska, 2017). However, we found less evidence of purely professional notions of shared values and approaches to practice, though this may be a consequence of our analysis arising from a survey focused on the implementation of revalidation, a regulatory process.

Our findings reveal, therefore, that ROs' role in relation to the profession represents structural restratification (Waring, 2014), but that the discourses which frame their work draw more upon managerial and regulatory logics than those emanating from within the profession. Although the RO role preserves a core regulatory function for qualified doctors, the surveillance of the profession they undertake serves to facilitate the advancement of agendas largely determined externally, rather than bolstering professional autonomy in any meaningful way. Indeed, ROs' hybrid nature, necessitated by their position at the confluence of three co-existing logics (McDonald et al., 2013), means that analyses suggesting homogenous resistance to regulatory power (Spendlove, 2018) ignore the nuances of professional strata.

Our study contributes to the literature on hybrid healthcare professionalism. Existing studies on restratification and hybridity have typically focused on those in doctor-manager positions – the managerial elite – where the focus of their work is on delivering healthcare services (Kuhlmann et al., 2013; McGivern et al., 2015). Our research evidences for the first time that ROs' regulatory responsibilities bring a distinct dimension to their experiences and to their emergent group identity. Whilst many ROs hold an MD role too, our analysis indicates that the regulatory focus of RO work, especially making revalidation recommendations, distinguishes it from the doctor-manager work studied elsewhere. Our analysis extends understandings of professional hybridity, moving beyond binary ‘medical/managerial’ hybrids to look at the complex interplay of multiple intra- and extra-professional agendas. The convergence of professional hierarchy, managerial status, and regulatory responsibilities that define the RO role point to the emergence of a distinctly regulatory form of hybrid professionalism.

Whilst recognising the limitations of survey data, particularly the brevity of some responses, and the lack of opportunity for immediate follow-up, in this instance the high response rate yielded rich qualitative data for analysis. This analysis has, however, highlighted a number of avenues that warrant further in depth exploration, including notably some of the professional, contextual and social factors underlying RO behaviour. For example, how and why individuals come to take on elite professional roles is worthy of further attention. Likewise, research could shed more light on the issue of RO identity formation, at both individual and group levels. However, our findings also suggest that some ROs felt that their regulatory tasks detracted from other areas of their work, particularly clinical responsibilities. For many, RO work is only one element of a portfolio of roles held concurrently, which may encompass clinical, educational, or wider managerial aspects; and our findings arise from a questionnaire particularly focused on the regulatory dimension. We might, therefore, seek to better understand how continuing to practise clinically whilst overseeing others' medical practice impacts on the formation of hybrid professional identity. Finally, whilst our focus here has been solely on ROs' experiences from their own perspective, a more rounded understanding of this group would result from bringing in the voices of those that they oversee, and their colleagues in organisational management, including other doctor-managers and non-clinical colleagues.

## 4 Conclusion

Our findings highlight the importance of ROs as a distinct group of hybrid professionals, forming a governance elite and a new locus of power in UK medicine. However, whilst the emergence of this group represents a restructuring of the medical profession, the regulatory focus of their activities and the lines of power and accountability between the wider profession and the GMC that operate through them, suggest that the RO function is not one which primarily acts in defence of professional autonomy. Rather, ROs' work, and their attitudes towards fulfilling their core task of monitoring other doctors' fitness to practise, seem likely to expand professional regulation into the organisational sphere in new ways. Understanding ROs' developing role at the confluence of regulatory, organisational and professional agendas offers an opportunity to explore the on-going development of professionalism in an era of strengthened governance and complex policy change.

## Funding

This paper is an output from independent research commissioned and funded by the Department of Health Policy Research Programme (PR-R9-0114-11002: Evaluating the development of medical revalidation in England and its impact on organisational performance and medical practice). The views expressed in this publication are those of the author(s) and not necessarily those of the Department of Health. The funder had no role in study design; in the collection, analysis and interpretation of data; in the writing of the articles; nor in the decision to submit it for publication.
